# Longitudinal Blood‐Biomarker‐Based Assessment of Brain Injury in Patients Undergoing Deep Brain Stimulation and Magnetic Resonance–Guided Focused Ultrasound

**DOI:** 10.1002/mds.70071

**Published:** 2025-09-30

**Authors:** Justina Dargvainiene, Ann‐Kristin Helmers, Juliana Naumann, Carl Alexander Gless, Bettina Möller, Julius Welzel, Frank Leypoldt, Johannes Hensler, Ina Tesseur, Klaus‐Peter Wandinger, Günther Deuschl, Daniela Berg, Steffen Paschen

**Affiliations:** ^1^ Institute of Clinical Chemistry University Medical Center Schleswig‐Holstein Kiel Germany; ^2^ Department of Neurosurgery University Hospital Schleswig‐Holstein, Christian‐Albrechts‐University Kiel Germany; ^3^ Department of Neurology University Hospital Schleswig‐Holstein, Christian‐Albrechts‐University Kiel Germany; ^4^ Department of Radiology and Neuroradiology University Hospital Schleswig‐Holstein, Christian‐Albrechts‐University Kiel Germany; ^5^ UCB Braine‐l'Alleud Belgium

**Keywords:** deep brain stimulation, glial fibrillary acidic protein (GFAP), magnetic resonance–guided focused ultrasound (MRgFUS), neurofilament light chain (NfL)

## Abstract

**Background:**

Deep brain stimulation (DBS) and magnetic resonance–guided focused ultrasound (MRgFUS) are associated with neuroaxonal damage and astroglial activation; yet their extent and timing remain unclear despite clinical relevance for monitoring and outcome assessment.

**Objective:**

This study assessed neuroaxonal damage and astroglial activation after DBS (n = 21) and MRgFUS (n = 19) reflected by serum neurofilament light chain (sNfL) and serum glial fibrillary acidic protein (sGFAP).

**Methods:**

Samples were collected at baseline, 24 h, 7 days, and 3/6/9 months posttreatment. Biomarker levels were measured using a single‐molecule array (Simoa).

**Results:**

sNfL peaked at day 7 and sGFAP at 24 h post‐intervention in both groups. sNfL normalized at 6 months in DBS and 3 months in MRgFUS. sGFAP normalized within 7 days in both groups. Biomarker elevations were higher in DBS patients.

**Conclusions:**

DBS and MRgFUS cause transient neuroaxonal injury and astroglial activation, with greater extent in DBS. Biomarker monitoring suggests final clinical evaluation should be performed 6 months after treatment at the earliest. © 2025 The Author(s). *Movement Disorders* published by Wiley Periodicals LLC on behalf of International Parkinson and Movement Disorder Society.

Deep brain stimulation (DBS), and more recently magnetic resonance–guided focused ultrasound (MRgFUS), is an emerging surgical treatment for movement disorders, including Parkinson's disease (PD),^1^, ^2^ essential tremor (ET),^3^, ^4^ and dystonia.^5^ These therapies have demonstrated promising clinical outcomes, leading to a reduction in pharmacological interventions and an overall improvement in quality of life. However, both modalities are associated with some degree of mechanical/thermal injury to the central nervous system. Data on the extent and dynamics of this localized neural tissue damage post‐intervention are scarce.^6^

The aim of our study was to longitudinally assess neuroaxonal and astroglial damage in patients undergoing DBS and MRgFUS. We analyzed two established blood‐based biomarkers: neurofilament light chain (NfL), a marker of neuroaxonal injury, and glial fibrillary acidic protein (GFAP), a marker of astrocytic activation and damage. Both biomarkers are known to be quickly elevated after injury to the central nervous system and to exhibit detectable changes not only in the cerebrospinal fluid but also in the blood.^7^, ^8^, ^9^ The analysis of NfL and GFAP thus allows neuronalaxonal and glial damage to be detected, quantified, and tracked over time, leading to better risk assessment, treatment planning, and follow‐up care.

## Methods

### Participants and Study Design

Between March and November 2023, 56 patients planned for DBS surgery and MRgFUS treatment were screened for study participation. Patients were included in the study if they met the following inclusion criteria: (1) diagnosis of PD, dystonia, or ET according to the MDS diagnosis criteria/tremor classification^10^, ^11^, ^12^ and (2) indication for DBS or MRgFUS. Exclusion criteria were (1) diagnosis of a neurodegenerative disease other than PD; (2) history of brain injury, brain atrophy, abnormal bleeding, and cerebrovascular and cerebral inflammatory disease; (3) missing follow‐up data; or (4) dropout before end of study at month 9.

### Visits, Sample Collection, and Measurements

Samples were collected at baseline (1 day before and on the morning of treatment), 24 h posttreatment, 7 days posttreatment, and at 3, 6, and 9 months using serum monovettes (Sarstedt, Nümbrecht, Germany). Samples from our clinic were centrifuged at 2500 rpm for 10 min and stored at −80°C within 4 h. When in‐person visits were not feasible, follow‐up samples were obtained by general practitioners and shipped to our laboratory, with sampling and processing dates recorded. This approach was based on data confirming NfL stability at room temperature for up to 7 days.^13^

All samples were analyzed using a single‐molecule array (Simoa, Billerica, Massachusetts, USA) Neurology 2‐Plex assay (NfL, GFAP) on a fully automated HD‐X Analyzer (Quanterix). Measurements were performed in two batches with randomized samples. Clinical data included demographics and disease severity scores before and after treatment. In DBS patients, the number of microelectrodes was documented, whereas in MRgFUS patients treatment details (ie, applied energy and skull density ratio) were recorded.

Post MRgFUS treatment lesion volume (mm^3^) was studied as described before (Data S1).^14^, ^15^

### Statistical Analysis

Baseline data were calculated by evaluating the mean of blood samples taken the day before and the morning of DBS or MRgFUS. Normal distribution of data was controlled using Shapiro–Wilk's test. Group comparisons were performed using repeated‐measures analysis of variance (ANOVA) and post hoc testing. *P*‐values were adjusted using Holm correction. Violations in the assumptions required for an ANOVA were controlled, and if needed Greenhouse–Geisser sphericity correction was applied. ω^2^ effect size calculation was used to provide an unbiased effect size measure. Correlation analysis was performed using Pearson's or Spearman's correlation analysis, and *P*‐values <0.05 were considered statistically significant. Statistical analysis was performed using JASP‐Team (version 0.18/University of Amsterdam/Netherlands).

## Results

Fifty‐six patients received DBS (n = 30) or MRgFUS (n = 26) treatment. Of these, 40 patients met the inclusion/exclusion criteria (DBS: n = 21, MRgFUS: n = 19; see flowchart Fig. S1). Baseline characteristics of the patients, including disease‐specific scores (Movement Disorders Society‐modified Unified Parkinson's Disease Rating Scale, Unified Dystonia Rating Scale, The Essential Tremor Rating Assessment Scale), are summarized in Table 1 and Table S2. Patients in the MRgFUS group were older and had higher baseline serum neurofilament light‐chain (sNfL) levels compared to those in the DBS group. At baseline, sNfL—but not sGFAP—exhibited a strong correlation with age (r = 0.526, *P* < 0.001).

**TABLE 1:** Patient baseline characteristics

	DBS	FUS	*P*‐value
Number of patients	21	19	
Male/female, N (%)	11/10 (52/48)	16/3 (84/16)	
Age (y, mean ± SD)	63.2 ± 6.7	72.1 ± 9.3	0.003**
Disease, N (%)			
ET	3 (14%)	9 (47%)	
PD	15 (71%)	8 (42%)	
Dystonia	3 (14%)	2 (11%)	
NfL (pg/mL, mean ± SD)	15.4 ± 6.5	22.2 ± 8.2	0.005**
GFAP (pg/mL, mean ± SD)	174.0 ± 82.4	235.9 ± 167.8	0.27

Abbreviations: DBS, deep brain stimulation; FUS, focused ultrasound; SD, standard deviation; ET, essential tremor; PD, Parkinson's disease; NfL, neurofilament light chain; GFAP, glial fibrillary acidic protein.

* *P* < 0.05;

** *P* < 0.01;

*** *P* < 0.001.

**FIG. 1:**
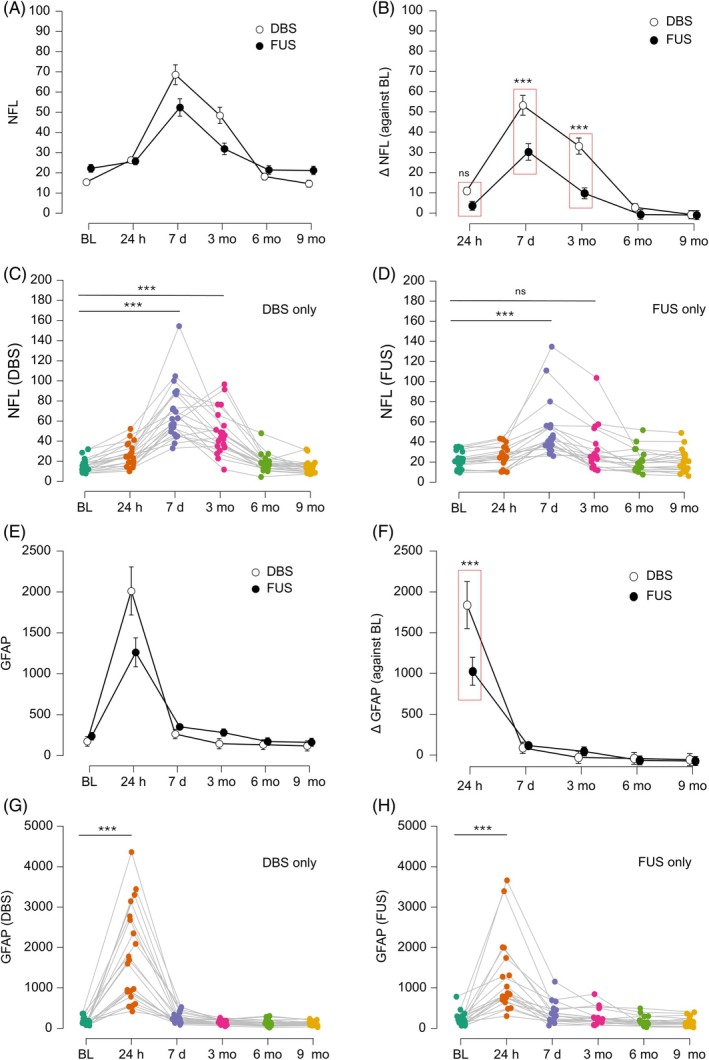
Time course of serum neurofilament light chain (sNfL) and glial fibrillary acidic protein (GFAP) after magnetic resonance–guided focused ultrasound (MRgFUS) and deep brain stimulation (DBS) treatment. The time course and extent of NfL and GFAP increases after DBS surgery and MRgFUS treatment are shown. (**A**, **E**) The mean ± SE (standard error of the mean) for NfL and GFAP, respectively. (**B**, **F**) The difference in NfL and GFAP levels relative to baseline. (**C**, **D**, **G**, **H**) The time course for individual patients, split into DBS and MRgFUS groups, for NfL and GFAP, respectively. The NfL increase was highest after 7 days (A) and normalized after 6 months in the DBS group (C) and 3 months in the MRgFUS group (D). GFAP levels peaked after 24 h (E) and normalized 7 days after treatment in both groups (G, H). Increases in NfL and GFAP were higher in DBS than in MRgFUS treatments (B, F). The data in fields (A, B, E, F) are given as mean ± SD (standard deviation). All NfL and GFAP values are expressed in picograms per milliliter. [Color figure can be viewed at wileyonlinelibrary.com]

Peak sNfL concentrations were observed in both the DBS (68.5 ± 28.2 pg/mL) and MRgFUS (52.3 ± 28.2 pg/mL) group 7 days posttreatment (Fig. 1A; Table S1), whereas peak sGFAP concentrations were reached at 24 h posttreatment (2011.2 ± 1513.1 pg/mL in DBS and 1261.2 ± 933.1 MRgFUS) (Fig. 1E; Table S1). sNfL levels returned to baseline at 6‐month follow‐up in DBS patients, whereas in the MRgFUS group, sNfL levels were not significantly different at the 3‐month follow‐up (Fig. 1C,D; Table S1). sGFAP levels returned to baseline at the 7‐day follow‐up in both DBS and MRgFUS patients (Fig. 1G,H; Table S1). Changes in sNfL and sGFAP from baseline (ΔNfL and ΔGFAP) were greater in DBS patients compared to the MRgFUS group (Fig. 1B,F; Table S1). In addition, the increase in sNfL 7 days after treatment was highly correlated with the increase in sGFAP 24 h after treatment (r = 0.425, *P* = 0.006). The increase in neither sNfL nor sGFAP was correlated with age, underlying disease, improvement in the respective disease‐specific scores, number of side effects, number of inserted microelectrodes in DBS surgery or skull density ratio, number of sonications, maximum temperature, total energy applied, or lesion size in FUS treatment (Tables S2 and S3). However, lesion size correlated with the number of sonications >54°C (r = 0.741, *P* = 0.001).

## Discussion

The main findings of our study include elevated sNfL levels in both DBS and MRgFUS patients, persisting for up to 6 months, with peak concentrations observed 7 days posttreatment. Additionally, sGFAP levels were elevated in both DBS and MRgFUS patients, peaking at 24 h posttreatment and returning to baseline within 7 days.

Previous studies investigating cerebrospinal fluid (CSF) NfL levels after subthalamic nucleus stimulation in patients with PD have reported findings similar to ours, showing an increase in NfL concentrations within the first 2 weeks posttreatment, persistence of elevated levels for 3 to 6 months, and normalization beyond 12 months.^16^, ^17^ However, these studies were based on small cohorts with heterogeneous sampling intervals, leading to significant methodological limitations. To improve feasibility, we analyzed serum samples for greater accessibility and standardized sampling. Existing literature also supports serum‐based assessments due to a strong serum–CSF NfL correlation.^18^, ^19^ Two other studies investigating sNfL dynamics after subthalamic nucleus stimulation in PD reported a similar increase in sNfL levels persisting for 6 to 8 weeks post‐procedure, with a return to baseline at either 6^6^ or 12 months.^20^ Although these findings align with our observations, they do not account for sNfL dynamics at the 3‐month follow‐up. Data on neuroaxonal damage from MRgFUS are even more limited, but elevated NfL levels are expected due to thermal lesions detectable on MRI.^21^ A small study investigating MRgFUS in patients with Alzheimer's disease reported increased plasma and CSF NfL levels with a return to baseline 6 months posttreatment.^22^

Considering our findings alongside previous studies, current evidence suggests that NfL levels remain elevated in both CSF and blood for up to 3 to 6 months after microelectrode implantation in DBS treatment or MRgFUS. This prolonged elevation may indicate ongoing neuroaxonal damage during this period. However, the interpretation of sNfL as a biomarker of sustained neuroaxonal injury remains challenging, as its half‐life has not been experimentally determined. However, mathematical modeling estimates the half‐life of NfL in blood after a single‐hit event, such as traumatic brain injury, to be ~500 h (20 days).^23^ Consistent with our findings, studies on single‐hit events, such as stroke or traumatic brain injury, report sNfL elevation for weeks to months post‐injury, followed by a gradual return to baseline over months to years.^24^, ^25^, ^26^, ^27^ Therefore, it is most likely that both procedures (DBS and MRgFUS) represent a single‐hit event with gradual recovery of axonal damage for up to 6 months. As far as peak levels of NfL concentration can be compared considering different pathomechanisms of neuroaxonal damage, sNfL peak levels in DBS (48.4 ± 22.0 pg/mL) and MRgFUS patients (31.9 ± 22.5 pg/mL) were lower compared to patients with ischemic stroke (91.34 ± 72.2 pg/mL)^24^ and traumatic brain injury (507.3 ± 728.2 pg/mL).^28^

In relation to the changes in sGFAP levels observed in our study, GFAP is widely recognized as a biomarker of astrocytic activation and injury. In conditions without significant astrogliosis or scar formation, such as traumatic brain injury, the half‐life of GFAP has been reported to be considerably shorter than that of sNfL, typically ranging from 24 to 72 h.^29^, ^30^ Conversely, in less acute conditions characterized by persistent neuroinflammation, GFAP levels have been reported to remain elevated for weeks or longer, likely reflecting sustained astrocytic activation.^31^ The mechanisms of injury and the temporal dynamics of sGFAP levels observed in our study indicate an acute condition without subsequent significant astrogliosis. This aligns with histopathological studies in DBS patients, showing a thin GFAP‐positive capsule around the DBS electrode, due to electrode placement, not electrical stimulation.^32^

Second, we observed greater changes from baseline in both sNfL and sGFAP in the DBS group compared to the MRgFUS group. Thus, it is tempting to speculate that the axonal and astrocytic injury is more pronounced in DBS surgery compared to MRgFUS treatment. However, these results should be interpreted with caution, as several covariates affecting baseline biomarker levels, such as age and underlying disease, differed between the two groups.^33^

As both treatment groups showed a return to baseline at a similar time point, it is very likely that the nature of the injury is comparable between the two groups, although the injury in DBS is due to DBS electrode implantation and the lesion in MRgFUS is due to thermal cell damage from ultrasound application.

We explored the relationship between changes in sNfL and sGFAP, clinical outcome, and treatment parameters (eg, electrode count in DBS, sonications in MRgFUS, lesion volume) but found no significant associations. This may be due to the small sample size, a key study limitation.

## Conclusions

Our study results suggest that MRgFUS and DBS cause transitory neuroaxonal damage and astroglial activation/damage that can be monitored using serum‐based biomarkers and remain elevated over the period up to 6 months. This finding indicates that the final clinical evaluation should be assessed after this time period, as prolonged neuroaxonal damage may occur during this period. The change from baseline (ΔNfL and ΔGFAP) was greater in DBS patients, indicating greater axonal and astrocytic damage after DBS surgery compared to MRgFUS treatment.

## Author Roles

(1) Research project: A. Conception, B. Organization, C. Execution, D. Magnetic resonance imaging and technical data analysis; (2) Statistical analysis: A. Design, B. Execution, C. Review and critique; (3) Manuscript preparation: A. Writing of the first draft, B. Review and critique.

J.D.: 1A, 1B, 1C, 2A, 2C, 3A, 3B

A.‐K.H.: 2C, 3B

J.N.: 1D, 2C, 3B

C.A.G.: 1D, 2C, 3B

B.M.: 2C, 3B

J.W.: 2C, 3B

F.L.: 1A, 1B, 2C, 3B

J.H.: 1D, 2C, 3B

I.T.: 1A, 2C, 3C

K.‐P.W.: 2C, 3B

G.D.: 2B, 3B

D.B.: 1A, 1B, 2C, 3B

S.P.: 1A, 1B, 1C, 2A, 2B, 2C, 3A, 3B

## Full financial disclosures of all authors for the preceding 12 months

J.D. reports no disclosures. A.‐K.H. received lecture fees from Boston Scientific and Insightec outside the submitted work. J.N. reports no disclosures. C.A.G. reports no disclosures. B.M. reports no disclosures. J.W. reports no disclosures. F.L. is funded by grants from the German Federal Ministry of Education and Research (CONNECT‐GENERATE grant numbers 01GM1908A and 01GM2208). F.L. is also supported by E‐Rare Joint Transnational research support (ERA‐Net, LE3064/2‐1) and Stiftung Pathobiochemie of the German Society for Laboratory Medicine and HORIZON MSCA 2022 Doctoral Network 101119457—IgG4‐TREAT and discloses speaker honoraria from Grifols, Teva, Biogen, Bayer, Roche, Novartis, and Fresenius; travel funding from Merck, Grifols, and Bayer; and advisory board fees for Roche, Biogen, and Alexion. J.H. has served as a consultant for Stryker Neurovascular and BALT. He has received reimbursement of travel expenses to attend scientific meetings by Rapid Medical outside the submitted work. I.T. is a full‐time employee of UCB Biopharma SRL. K.‐P.W. reports no disclosures. G.D. has served as a consultant for Boston Scientific, Cavion, and Functional Neuromodulation. He has received royalties from Thieme Publishers and funding from the German Research Council (SFB 1261, T1) and private foundations. D.B. received consultancies/advisory board fees from UCB Pharma GmbH, and grants/research funding from Deutsche Forschungsgemeinschaft (DFG), German Parkinson's Disease Association (dPV), BMBF, Parkinson Fonds Deutschland gGmbH, UCB Pharma GmbH, EU, Novartis Pharma GmbH, the Damp Foundation, Biohaven, BMBF, Else Kröner‐Stiftung, Hoffmann La Roche AG, Jan von Appen Stiftung, Lundbeck, and The Michael J. Fox Foundation (MJFF). S.P. reports speaker honoraria from Insightec, AbbVie, Medtronic GmbH, and Boston Scientific outside the submitted work and grant/research funding from Deutsche Forschungsgemeinschaft (DFG), Parkinson Fonds Deuschland gGmbH, and UCB Pharma GmbH.

## Supporting information


**Figure S1.** Study flow chart.

**Table S1.** Time course and extent of NfL and GFAP after DBS surgery and MRgFUS treatment.
**Table S2.** Details on essential tremor, Parkinson disease and dystonia patients.
**Table S3.** DBS and FUS procedure.

## Data Availability

The data that support the findings of this study are available on request from the corresponding author. The data are not publicly available due to privacy or ethical restrictions.
