# Supplementary figures and images for: Longitudinal Blood‐Biomarker‐Based Assessment of Brain Injury in Patients Undergoing Deep Brain Stimulation and Magnetic Resonance–Guided Focused Ultrasound
Source: Mov Disord. 2025 Sep 30;41(1):241–6. doi: 10.1002/mds.70071 (PMC12882050; doi:10.1002/mds.70071)

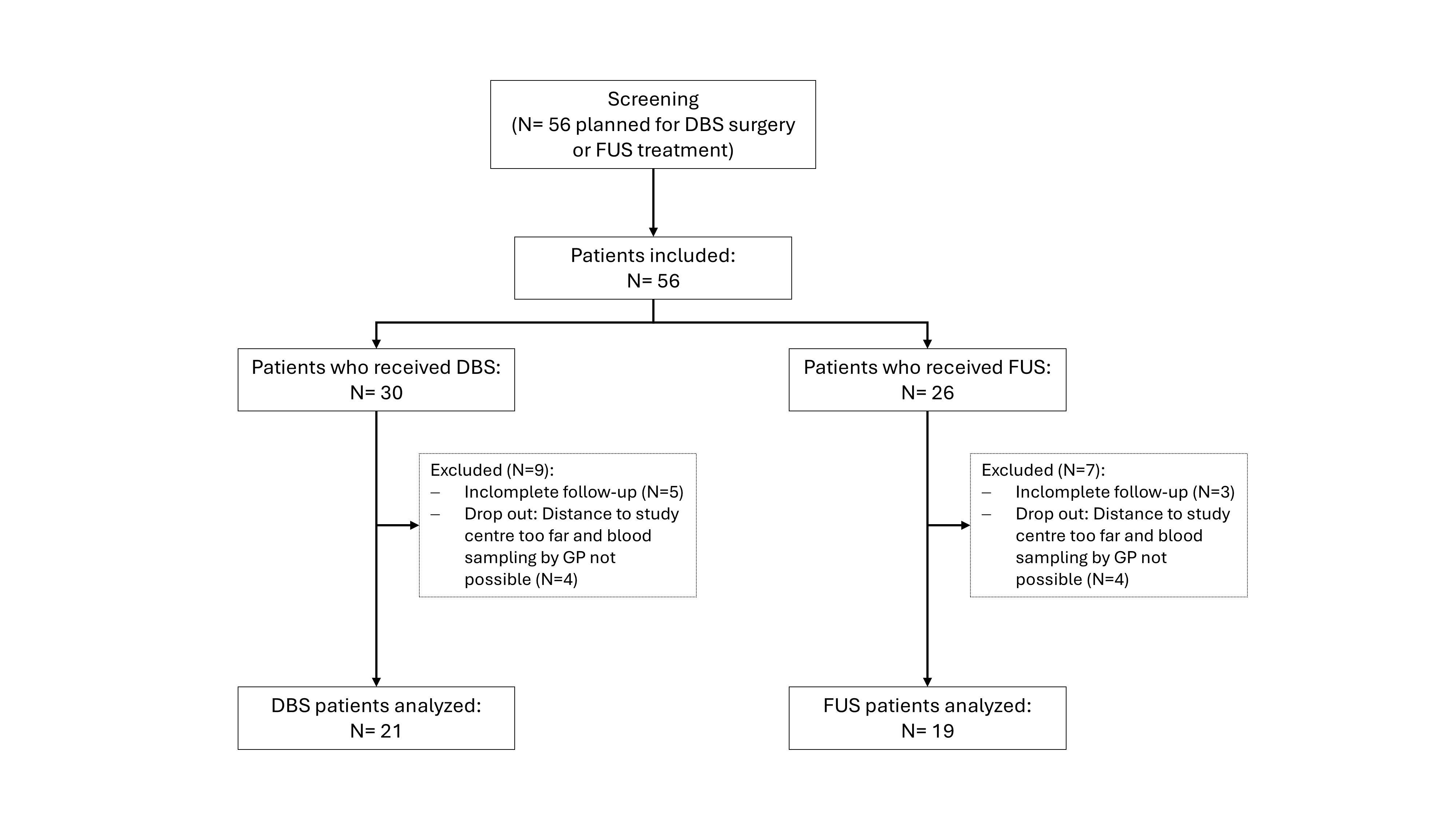

Supplement: Supplementary file 1 — Figure S1. Study flow chart. [file MDS-41-241-s002.tif]
