# Supplementary material for: Longitudinal Blood‐Biomarker‐Based Assessment of Brain Injury in Patients Undergoing Deep Brain Stimulation and Magnetic Resonance–Guided Focused Ultrasound
Source: Mov Disord. 2025 Sep 30;41(1):241–6. doi: 10.1002/mds.70071 (PMC12882050; doi:10.1002/mds.70071)
Supplement: Supplementary file 2 — Table S1. Time course and extent of NfL and GFAP after DBS surgery and MRgFUS treatment. Table S2. Details on essential tremor, Parkinson disease and dystonia patients. Table S3. DBS and FUS procedure. [file MDS-41-241-s001.docx]

Suppl. material Table 1: Time course and extent of NfL and GFAP after DBS surgery and MRgFUS treatment

| Timepoint |  | **NFL (pg/ml)** | | | | **GFAP (pg/ml)** | | | |
| --- | --- | --- | --- | --- | --- | --- | --- | --- | --- |
|  |  | **Within group comparison** | | **Between group comparison** | | **Within group comparison** | | **Between group comparison** | |
|  |  | **Mean**  ±SD | **P value** (against BL) | **Delta** (against BL)  ±SD | P value (DBS vs. FUS) | **Mean**  ±SD | **P value** (against BL) | **Delta** (against BL)  ±SD | P value (DBS vs. FUS) |
| Baseline | DBS | 15.4  ±6.5 |  | | | 174.0  ±82.4 |  | | |
|  | FUS | 22.2  ±8.2 |  |  |  | 235.9  ±167.8 |  |  |  |
| 24 hours | DBS | 26.3  ±11.3 | 0.177 | 10.9  ±7.1 | 1.000 | 2011.2  ±1513.1 | < .001^***^ | 1837.2  ±1472.3 | < .001*** |
|  | FUS | 25.7  ±10.5 | 1.000 | 3.5  ±3.6 |  | 1261.2  ±933.1 | < .001^***^ | 1025.3  ±869.6 |  |
| 7 days | DBS | 68.5  ±28.2 | < .001^***^ | 53.2  ±23.8 | < .001*** | 262.9  ±133.3 | 1.000 | 88.9  ±102.8 | 1.000 |
|  | FUS | 52.3  ±28.2 | < .001^***^ | 30.2  ±24.1 |  | 352.0  ±259.4 | 1.000 | 116.1  ±197.1 |  |
| 3 months | DBS | 48.4  ±22.0 | < .001^***^ | 33.0  ±19.7 | < .001*** | 144.9  ±54.8 | 1.000 | -29.1  ±56.6 | 1.000 |
|  | FUS | 31.9  ±22.5 | 0.078 | 9.7  ±7.2 |  | 280.5  ±195.2 | 1.000 | 44.6  ±69.0 |  |
| 6 months | DBS | 18.1  ±8.7 | 1.000 | 2.8  ±5.9 | 1.000 | 132.8  ±72.6 | 1.000 | -41.2  ±50.5 | 1.000 |
|  | FUS | 21.4  ±11.3 | 1.000 | -0.8  ±8.9 |  | 170.8  ±122.3 | 1.000 | -65.1  ±174.1 |  |
| 9 months | DBS | 14.6  ±6.5 | 1.000 | -0.8  ±4.0 | 1.000 | 117.1  ±52.9 | 1.000 | -56.9  ±68.4 | 1.000 |
|  | FUS | 21.2  ±11.3 | 1.000 | -1.0  ±8.7 |  | 162.3  ±106.8 | 1.000 | -73.6  ±171.0 |  |
| The table shows the time course of NfL and GFAP compared to the baseline value within each group, as well as the relative increase in NfL and GFAP compared to baseline, with a comparison between DBS and MRgFUS.  Data are given as mean±SD. All NfL and GFAP values (within and between group comparisons) are expressed in pg/mL.  *P<0.05, **P<0.01, ***P<0.001. NFL = neurofilament light chain, GFAP = glial fibrillary acidic protein. | | | | | | | | | |

Suppl. Table 2: Details on essential tremor, Parkinson disease and dystonia patients

|  | DBS | | FUS | | *P* value  (between group comparison) |
| --- | --- | --- | --- | --- | --- |
| Essential tremor patients | n=3 | | n=9 | |  |
| Gender, male | 1/3 | 33% | 7/9 | 78% |  |
| Age (years) | 63.7 | ±10.2 | 73.1 | ±10.7 | 0.211 |
| TETRAS, total, baseline | 55.0 | ±5.5 | 56.4 | ±10.2 | 0.829 |
| TETRAS, total, 3 months FU | n.a. | n.a. | 40.0 | ±14.6 |  |
| Target:  Vim/Thalamotomy | 3/3 | 100% | 9/9 | 100% |  |
| NFL increase 7 days after treatment (pg/ml) | 48.7 | 11.0 | 29.5 | 19.9 | 0.1 |
| GFAP increase 24 hours after treatment (pg/ml) | 2202.5 | 1511.6 | 970.7 | 858.7 | 0.282 |
|  | | | | | |
| Parkinson’s disease patients | n=15 | | n=8 | |  |
| Gender, male | 10/15 | 67% | 7/8 | 88% |  |
| Age (years) | 63.3 | ±6.3 | 71.0 | ±8.4 | 0.021* |
| MDS-UPDRS, part III, med. off, baseline | 45.1 | ±18.8 | 47.3 | ±5.2 | 0.765 |
| MDS-UPDRS, part III, med. on, 3 months FU | 19.9 | ±13.0 | 25.5 | ±10.9 | 0.215 |
| Target:  Vim/Thalamotomy  STN/Subthalamotomy | 0/15  15/15 | 0%  100% | 6/8  2/8 | 75%  25% |  |
| NFL increase 7 days after treatment (pg/ml) | 54.4 | ±27.4 | 32.5 | ±31.6 | 0.024* |
| GFAP increase 24 hours after treatment (pg/ml) | 1703.3 | ±1563.5 | 1014.3 | ±999.7 | 0.213 |
|  | | | | | |
| Dystonia patients | n=3 | | n=2 | |  |
| Gender, male | 0/3 | 0% | 2/2 | 100% |  |
| Age (years) | 62.3 | ±8.1 | 71.5 | ±12.0 | 0.400 |
| UDRS, total, baseline | 22.5 | ±8.5 | n.a. | n.a. |  |
| TETRAS, total, baseline | n.a. | n.a. | 56.3 | ±18.0 |  |
| TETRAS, total, 3 months FU | n.a. | n.a. | 26.5 | ±24.8 |  |
| Target:  Vim/Thalamotomy  GPi/Pallidotomy | 0/3  3/3 | 0%  100% | 2/2  0/2 | 100%  0% |  |
| NFL increase 7 days after treatment (pg/ml) | 51.3 | ±15.8 | 23.9 | ±18.6 | 0.4 |
| GFAP increase 24 hours after treatment (pg/ml) | 2141.4 | ±1344.2 | 1314.7 | ±721.8 | 0.8 |
| The table shows a detailed analysis of the patients included, separated by underlying disease (essential tremor, Parkinson's disease, and dystonia). For each disease, the baseline characteristics, including the main disease severity scores and treatment targets, as well as the increase in NfL and GFAP at 7 days and 24 hours, were compared.  Disease severity was assessed in the standardized, clinical routine assessments for essential tremor patients using the Essential Tremor Rating Assessment Scale (TETRAS). For patients with Parkinson's disease, the Movement Disorder Society-Unified Parkinson's Disease Rating Scale (MDS-UPDRS) was utilized in both the medication off (med. off) and medication on (med. on) states. Dystonia patients were assessed using the Unified Dystonia Rating Scale (UDRS) and TETRAS. between DBS and MRgFUS patients.  Data are given as mean±SD. **P*<0∙05, ***P*<0∙01, ****P*<0∙001. GFAP = glial fibrillary acidic protein, NFL = neurofilament light chain, GPi = Globus pallidus internus, STN = subthalamic nucleus, Vim=ventral-intermediate nucleus of thalamus. | | | | | |

Suppl. Table 3: DBS and FUS procedure

|  | | | **Correlation analysis** | | | |
| --- | --- | --- | --- | --- | --- | --- |
|  | | | Δ NFL increase 7 days after treatment | | Δ GFAP increase 24 hours after treatment | |
| **DBS** | | | Spearman’s rho | P value | Spearman’s rho | P value |
| Number of microelectrodes | 2.8 | ±0.8 | -0.015 | 0.950 | 0.057 | 0.805 |
|  | | |  | | | |
| **FUS** | | | Spearman’s rho | P value | Spearman’s rho | P value |
| Treated hemisphere:  Right  Left | 5  14 |  |  |  |  |  |
| Skull density ratio | 0.52 | ±0.1 | 0.131 | 0.593 | 0.225 | 0.355 |
| Number of sonications  Number of Sonications ≥ 54 C | 8.0  2.6 | ±1.7  ±1.4 | -0.044  0.164 | 0.858  0.503 | -0.215  0.359 | 0.376  0.132 |
| Total energy applied (Joule) | 83750 | ±57163 | -0.291 | 0.226 | -0.237 | 0.327 |
| Lesion volume (mm3) | 255.9 | ±130.3 | 0.160 | 0.526 | 0.318 | 0.198 |
| Data are given as mean (±SD). NFL = neurofilament light chain, GFAP = glial fibrillary acidic protein. | | | | | | |

Suppl. material:

MRgFUS lesion volume analysis:

Post MRgFUS-treatment lesion volume (mm3) was studied after manual segmentation on T1-w MRI acquired 24 hours after the procedure as described before.^14,15^ Briefly, lesion boundaries were defined using ITK-Snap segmentation software by identifying the lesion core and periphery according to Wintermark et al.^1^ All images were segmented by two independent raters, and only voxels labelled as lesions by both raters were included in the final segmentation. To assess inter-rater variability, the Kappa similarity coefficient (Cohen's Kappa) was calculated for all segmentations. The mean inter-rater kappa of 0.85 ± 0.04, calculated from these segmentations, indicates high reliability and minimal variability between raters in lesion identification.

1. Wintermark M, Druzgal J, Huss DS, et al. Imaging findings in MR imaging‐guided focused ultrasound treatment for patients with essential tremor. AJNR Am J Neuroradiol 2014;35(5):891–896.
